# Effects of honeysuckle vine powder on growth, immune performance, and gastrointestinal microbiota structure of Nanjiang Yellow goats

**DOI:** 10.3389/fmicb.2025.1727133

**Published:** 2026-01-14

**Authors:** Kerui Li, Lian Huang, Changhui Zhang, Hua Xiang, Kang Jiang, Wangsheng Zhao, Hengbo Shi, Jiangjiang Zhu, Zhanyu Du

**Affiliations:** 1 Qinghai-Tibetan Plateau Animal Genetic Resource Reservation and Utilization Key Laboratory of Sichuan Province, Chengdu, China; 2 Bazhong Academy of Agriculture and Forestry Sciences, Bazhong, Sichuan, China; 3 College of Life Sciences and Agri-forestry, Southwest University of Science and Technology, Mianyang, China; 4 Institute of Nanjiang Yellow Goat Sciences, Bazhong, Sichuan, China; 5 Institute of Dairy Science, College of Animal Sciences, Zhejiang University, Hangzhou, China

**Keywords:** honeysuckle vine powder, microbial restructuring, Nanjiang Yellow goats, production performance, serum immunoglobulin

## Abstract

**Introduction:**

Chinese herbal medicines, including honeysuckle (Lonicera japonica), exhibit diverse beneficial properties and are increasingly being explored as feed additives in livestock production to improve animal health and product quality. This study investigates the effects of honeysuckle vine powder (HVP) supplementation as an herbal feed additive on growth performance, immune function, and rumengut microbiota in Nanjiang Yellow goats during fattening.

**Methods:**

We evaluated four dietary groups: control (basal diet) and three experimental groups supplemented with 1% (EG1), 1.5% (EG2), and 2% HVP (EG3) for 60 days. After sampling, we measured the blood biochemical, immune indicators and carried out 16s RNA sequencing of rumen and gut microbiota for Nanjiang Yellow goat samples.

**Results:**

We evaluated four dietary groups: control (basal diet) and three experimental groups supplemented with 1% (EG1), 1.5% (EG2), and 2% HVP (EG3) for 60 days. Results demonstrated that 2% HVP significantly enhanced, average daily gain (ΔADG = 0.05 ± 0.02 /kg, *p* < 0.05, *n* = 8), and feed conversion rate (ΔFCR = 5.26 ± 0.69, *p* < 0.05, *n* = 8), while improving body length and chest circumference. Serum immunoglobulin (IgG, IgM) levels were elevated, indicating strengthened systemic immunity (*p* < 0.05). Critically, 16S rRNA analysis revealed HVP-induced rumen and gut microbiota restructuring, characterized by increased Firmicutes abundance.

**Discussion:**

These findings validate HVP as a sustainable feed additive that optimizes productivity and immune resilience through microbiota-host crosstalk, supporting its application in ecofriendly livestock farming.

## 1 Introduction

In recent years, driven by the ban on antibiotic use in livestock production and the growing demand for natural alternatives, Chinese herbal additives have emerged as a popular choice for feed supplements. This is attributed to their lack of contribution to drug resistance and diverse prebiotic functions. China possesses abundant resources of traditional Chinese medicine (Abdallah et al., 2019). When incorporated into animal diets, these botanical preparations serve dual roles in disease prevention and animal husbandry, thereby enhancing productive performance and product quality (Chen et al., 2023). Crucially, unlike conventional pharmaceuticals, they do not leave synthetic compound residues in tissues while exhibiting antibacterial and anti-inflammatory properties that improve meat quality, despite potential mild toxic effects (Pu et al., 2017; Wang et al., 2020). For instance, supplementation with *Lonicera japonica* Thunb. or its extract demonstrated significant inhibitory effects against pathogenic strains including Listeria and *Bacillus subtilis* (Rahman and Kang, 2009).

Numerous studies confirm that replacing artificial growth promoters and antibiotics with natural nutritional additives improves growth efficiency, immunity, and carcass characteristics in poultry and farm animals (Abd El-Hack et al., 2024). Specifically, dose-dependent positive correlations were observed between Echinacea powder extract (EPE) supplementation in broiler rations and weight gain. High-level EPE administration significantly enhanced growth parameters, strengthened immune function, and induced moderate hypolipidemic effects (Ashour et al., 2025). Similarly, *Paeonia lactiflora* extract administered to mice exhibited antioxidant, immunomodulatory, and anti-inflammatory activities (Su-Hong et al., 2015). Glycyrrhiza species have been documented for their bioactive properties including anti-inflammatory, antioxidant, and anticancer activities (Assar et al., 2021).

Honeysuckle (*Lonicera japonica*) and its by-products represent well-established components of traditional Chinese medicine. Substantial analyses reveal rich essential oil content, with 18 shared compound classes accounting for 85.23 and 83.42% of the total oils in flowers and stems, respectively. These essential oils act not only as growth promoters but also critically modulate rumen and intestinal microbiota dynamics while improving nutrient digestibility (Benchaar et al., 2008; Shang et al., 2011). Major organic acids include chlorogenic acid, isochlorogenic acid, and caffeic acid (Wu et al., 2012). Honeysuckle vine contains bioactive constituents primarily comprising phenolic acids, flavonoids, and terpenoids. Comparative HPLC-MS analyses confirm compositional similarities between the vine and its flower buds (Ye et al., 2014). Dietary supplementation with honeysuckle vine extract demonstrates significant potential in modulating rumen function and enhancing ruminant health. Studies indicate its efficacy in improving rumen fermentation parameters, reducing methane emissions, and boosting systemic antioxidant capacity and immune responses. For instance, supplementing heat-stressed dairy cows with 28 g/d of the extract effectively lowered rectal temperature while increasing serum glutathione peroxidase activity and IgG levels (Ma et al., 2020). Furthermore, inclusion at 1–2 g/kg DM in lactating cow diets increased dry matter intake and milk yield, concurrently reducing markers of inflammation and oxidative stress (Zhao et al., 2019). Post-harvest residues including branches, leaves, and vines are typically discarded despite their established use as medicinal materials (Shang et al., 2011; Kuroda et al., 2014) and potential value as feed additives for promoting growth, increasing body weight gain, and reducing serum lipid levels (Li Y. et al., 2023). Collectively, these findings highlight the promising role of honeysuckle vine extract as a functional feed additive for enhancing productivity and health in ruminant livestock systems. Similarly, studies in Holdobaki geese have shown that honeysuckle extract supplementation significantly improves both growth performance and immunological parameters (Li D. et al., 2023). Moreover, administering honeysuckle from late gestation through weaning was found to enhance antioxidant capabilities, liver health, colostrum quality, and immunity in sows, alongside improvements in their offspring’s growth and immune status (He et al., 2023).

In ruminants, rumen microbiota composition is intrinsically linked to growth performance, with the Firmicutes to Bacteroidota ratio (F/B ratio) widely recognized as a key indicator. Firmicutes enhance the degradation of fibrous substrates and promote short-chain fatty acid production, thereby boosting host energy harvest efficiency, while Bacteroidota primarily hydrolyze proteins and polysaccharides (Li S. et al., 2022; Wang et al., 2023). Higher F/B ratios positively correlate with improved feed conversion efficiency, average daily gain, and milk fat yield. For instance, dietary strategies increasing this ratio enhance body weight gain and nutrient digestibility in fattening cattle and lambs (Li J. et al., 2022). Furthermore, rumen microbial structure, and consequently the F/B ratio, can be modulated through dietary interventions, such as adjusting the concentrate to forage ratio or supplementing specific fatty acids to improve growth performance (Li et al., 2024; Vadroňová et al., 2024). However, the correlation between the F/B ratio and growth outcomes may vary depending on animal species, physiological stage, and feeding management systems, with some contradictory findings reported (Vadroňová et al., 2024). Overall, the F/B ratio represents a valuable microbial marker for ruminant nutritional metabolism and growth potential. Nevertheless, its precise regulatory mechanisms require further elucidation through more diversified functional studies of the rumen microbiome.

Herein, we conducted an experiment feeding diets containing 1, 1.5%, or 2% honeysuckle vine powder (HVP) to investigate its effects on growth performance, serum biochemical profiles, immune status, and gastrointestinal microflora composition in Nanjiang Yellow goats. We hypothesize that dietary formulations optimized with HVP incorporation may increase feed efficiency, while simultaneously enhancing immunity and growth performance via restructuring of ruminal and intestinal microbiota in goats.

## 2 Materials and methods

### 2.1 Experimental design and animal management

The study was carried out at the Original Breed Propagation Farm of Nanjiang Yellow goats in Bazhong City, Sichuan Province, China. Thirty-two three-month-old male goats with no genetic relationship to each other were randomly selected as experimental subjects. The inclusion criteria were an average weight of 20.38 ± 1.99 kg and having already been weaned. A significance analysis of the weight data was conducted prior to grouping the goats, to ensure no significant differences among experimental groups, within each group, and among replicates. The concentrate feed was procured from Advanced Feed Co., Ltd. The feed provided by this company was categorized into three types of supplementary feeds for fattening goat. The roughage was purchased on behalf of the breeding farm. The honeysuckle vine powder (HVP) was sourced from the local honeysuckle planting cooperative following the autumn pruning of honeysuckle vines. Subsequently, the fresh honeysuckle vines were placed in a forced-air drying oven and dried at 40 °C for 24 h. After that, they were ground and used. The passing rate through an 80-mesh sieve was ≥ 80%. The goats were randomly allocated into four groups (with eight goats in each group, and one enclosure for every two goats). Regarding cost-effectiveness, our study quantified the incorporation rate of honeysuckle vine powder in animal diets and optimized its dosage alongside other botanical additives. These groups were designated as the control group CG1 (fed a basal diet without honeysuckle vine powder, HVP), experimental group EG1 (supplemented with 1% HVP), experimental group EG2 (supplemented with 1.5% HVP), and experimental group EG3 (supplemented with 2% HVP). Each goat underwent preventive deworming, was weighed, and the floors and walls of the goat pens were disinfected. The goats were fed twice daily and had unrestricted access to fresh water. Following a 10-day pre-feeding period, the goats were fed with diets specific to their respective groups for 60 days, which served as the formal experimental period. The detailed nutritional composition of the commercial concentrated feed is presented in Supplementary Table S1.

### 2.2 Growth performance and sampling

Feed was supplied to each individual experimental goat at 08:00 and 17:00 daily. On the mornings of day 0, 15, 30, 45, and 60 during the formal feeding period, body weight, feed intake, and multiple body size metrics, such as heart girth, chest depth, height at withers, back height, body length, sacrum height, chest width, and cannon circumference were measured when the goats were in a fasting condition. To accurately determine average daily gain (ADG) and feed conversion ratio (FCR), all goats underwent a 12 h fasting period at both the initiation and completion of the trial before being weighed. Concurrently, blood samples (10 mL each) were drawn from the jugular vein into collection tubes without anticoagulants. The samples were allowed to clot at room temperature, followed by centrifugation at 2200 × g for 10 min at 4 °C to isolate the serum. The resulting serum was then carefully aliquoted using sterile pipettes and stored at −80 °C until further analysis. At the end of the 60-day feeding trial, all experimental goats underwent an 8 h fast. Subsequently, 50 mL of rumen fluid was collected from each animal. The collected fluid was filtered through sterile gauze to remove large particulates, then aliquoted into 5 mL cryovials and immediately immersed in liquid nitrogen for snap-freezing. Fecal samples were collected 1 day before the trial concluded. Hardened fecal pellets were aseptically obtained directly from the rectum using sterile tubes and subsequently stored at −80 °C until analysis.

### 2.3 Sample analyses

#### 2.3.1 Blood biochemical indicators and immune indicators determination

In our experiment, we conducted a hemolysis screening on all serum samples to ensure the quality of the samples. By observing the color of the serum or plasma with the naked eye, the results showed that no hemolysis occurred in any of the samples. Therefore, the impact of hemolysis on the experimental results can be disregarded. Serum samples were sent to Nanjing Jiancheng Hongda Biotechnology Co., Ltd. for analysis of immunoglobulin classes (H108-1-2 for IgA, H106-1-1 for IgG, H109-1-1 for IgM) and cytokines (H543-1), including interleukin (IL)-2 (H003-1-1), IL-4 (H005-1-2), and IL-6 (H007-1-1), as well as tumor necrosis factor alpha (H052-1-2 for TNF-*α*). Concurrently, other serum parameters were assessed in our own laboratory using commercial kits sourced from Nanjing Jiancheng Bioengineering Institute. Specifically, glucose levels were measured with a glucosoxidase assay (A154-1-1); total cholesterol was determined by a cholesterol oxidase–peroxidase coupled (COD-PAP) method (A111-1-1); and total protein concentration was quantified via the BCA method on microplates (A045-3-2). Furthermore, triglycerides were analyzed using a GPO-PAP kit (A110-1-1) following the urease procedure, and urea nitrogen levels were also determined (C013-2-1). All measurements were completed on a microplate reader (BioTek, United States). We conducted checks on the intra-assay coefficient of variation (Intra-assay CV) and inter-assay coefficient of variation (Inter-assay CV) with *n* = 5. After calculating the average values and standard deviations, the intra-assay coefficient of variation (Intra-assay CV) was all less than 5%, indicating that the experimental method has high precision within the same batch. The inter-assay coefficient of variation (Inter-assay CV) was all less than 10%, suggesting that the experimental method also has good precision between different batches. The results show that the experimental methods all have high reliability and repeatability.

#### 2.3.2 16S rRNA gene amplicon sequencing of rumen and gut microbiota

Samples were stored at −80 °C and subsequently thawed at 4 °C. Total DNA was extracted using the OMEGA Stool DNA Kit. We designed PCR primers targeting the conserved regions of the 16S rRNA gene to specifically amplify bacterial and archaeal sequences. Amplification was performed with the Phusion high-fidelity DNA polymerase. Following amplification, target fragments were purified using the AxyPrep PCR Cleanup Kit. The concentration of purified PCR products was measured using a Quant-iT PicoGreen dsDNA Assay Kit on a Promega QuantiFluor fluorometer. Based on these measurements, equimolar amounts of each sample’s PCR product were pooled to create a composite sequencing library. The resulting amplicon pool was submitted to Hangzhou Lianchuang Biotechnology Co., Ltd. for high-throughput sequencing on the Illumina Hiseq platform.

### 2.4 Bioinformatics and statistical analysis

Raw reads from 16S rRNA gene sequencing were processed using the QIIME2 pipeline. Briefly, raw reads were demultiplexed and quality-controlled using the qiime tools import and qiime demux emp-paired commands. Low-quality reads (those with a Phred quality score below Q20 or length shorter than 50 bp) were filtered out using the qiime quality-filter q-score command. Overlapping paired-end reads were merged into contiguous sequences using the qiime vsearch join-pairs command, requiring a minimum overlap of 10 bp and allowing no more than a 10% mismatch rate. Chimeric sequences were identified and removed using the qiime vsearch uchime-denovo command. Amplicon sequence variants (ASVs) were identified using the DADA2 plugin within QIIME2, which provides higher resolution compared to traditional 97% OTU clustering. Representative sequences for each ASV were taxonomically classified from phylum to species level against the SILVA reference database (version 138) using the qiime feature-classifier classify-sklearn command, with an assigned confidence cutoff of 0.8. Singleton ASVs (observed only once across all samples) were discarded. Instead of rarefaction, we used compositional methods provided by the q2-composition plugin to account for differences in sequencing depth and to avoid losing important biological information. Sequencing depth per sample ranged from 80,000 to 100,000 Raw tags (Supplementary Table S1). Negative controls were included in the sequencing run to monitor for contamination. No significant microbial signals were detected in the negative controls, indicating minimal contamination in the sequencing process.

Alpha diversity metrics, including observed richness (number of species), Chao1 index, Shannon index, Simpson index, and Good’s coverage, were estimated using QIIME2 v2023.2. Differences in alpha diversity among groups were consistently analyzed using the Kruskal-Wallis rank sum test, followed by *post hoc* pairwise comparisons with false discovery rate (FDR) correction for multiple testing. To identify taxa exhibiting significantly different abundances between groups, we applied two independent methods for cross-validation: (i) LEFSe analysis, considering features with an LDA score > 3.0 and an FDR-adjusted *p*-value < 0.05; and (ii) DESeq2 analysis, selecting features with |log₂ fold change| > 1 and an FDR < 0.05. Core microbiota members were defined as OTUs detected in at least 50% of all samples with a relative abundance of at least 0.1% within each sample where they were found. The majority of these bioinformatic analyses were executed on the OmicStudio cloud computing platform.^1^

All longitudinal data (body weight, feed intake, body size metrics, serum biomarkers, rumen/fecal indicators measured on days 0, 15, 30, 45, 60) were analyzed using linear mixed-effects models (LMMs) to account for repeated measures within the same goat. Fixed effects: Diet group, time point (categorical), and their interaction (diet × time). Random effect: Goat ID (random intercept). Covariance structure: An autoregressive (AR1) structure was selected based on Akaike Information Criterion (AIC) to model within-goat temporal correlations. Significant effects (*p* < 0.05) were further examined with Tukey’s HSD post-hoc tests. All *p*-values from multiple comparisons were adjusted via Benjamini-Hochberg false discovery rate (FDR) correction. Analyses were performed in R (v4.3.0) with “lme4” and “emmeans” packages.

## 3 Result

### 3.1 Animal growth performance

Growth performance results are summarized in Table 1. At both endpoints measured (15 and 30 days), average daily gain (ADG) in experimental groups EG3 were significantly higher than in the control group CG1 (*p* < 0.05). While no changes in body weight were detected among experimental groups at day 15, however, in the final result of 30 days, except for the CG1 group, all other groups showed an increasing trend but no have significantly. Over the subsequent month, this disparity became even more pronounced. Compared to CG1, EG1, and EG2, EG3 displayed significantly better FCR values (*p* < 0.05). Regarding physical measurements, the average growth in body length (AVGL) in the experimental groups surpassed that of CG1 (*p* < 0.05). Notably, the chest depth of the EG3 group was significantly greater than both CG1 and EG2 (*p* < 0.05). Similarly, chest circumference showed a significant (average chest growth, AVCG) increase in all experimental groups compared to the control (*p* < 0.05).

**Table 1:** Effects of dietary supplementation with HVP on growth performance in finishing goats.

Item	Grouping (*n* = 8/per group)			
	CG1	EG1	EG2	EG3
IW/kg	20.38 ± 1.99	20.31 ± 1.67	20.19 ± 1.16	20 ± 1.38
15d/kg	21.31 ± 2.25	22 ± 2.09	21.94 ± 1.37	21.86 ± 1.82
30d/kg	22.19 ± 2.59	23.06 ± 2.01	23.44 ± 1.82	23.34 ± 2.12
45d/kg	23.7 ± 3.15	25.13 ± 2.77	25.49 ± 2.17	24.9 ± 2.04
FW/kg	24.71 ± 3.68	26.95 ± 3.24	27.08 ± 2.08	27.3 ± 1.75
ADG/kg	0.08 ± 0.01^b^	0.09 ± 0.02^b^	0.09 ± 0.01^b^	0.13 ± 0.03^a^
ADG_95%_CI	0.06, 0.09^b^	0.07, 0.11^b^	0.07, 0.10^b^	0.12, 0.14^a^
AVGL/cm	3.06 ± 0.78^b^	5.44 ± 1.10^a^	5.06 ± 1.12^a^	5.71 ± 1.35^a^
AVGGH/cm	3.31 ± 1.30	4.13 ± 1.96	2.70 ± 2.05	3.42 ± 1.27
AVCG/cm	3.44 ± 1.27^b^	5.36 ± 0.92^a^	5.11 ± 0.77^a^	5.61 ± 1.31^a^
AVCDG/cm	2.06 ± 1.05^b^	2.56 ± 0.98^ab^	2.13 ± 0.83^b^	3.14 ± 0.90^a^
AVCWG/cm	1.81 ± 1.25	2.81 ± 0.84	2.94 ± 1.76	2.29 ± 1.22
ARHG/cm	4.38 ± 2.26	4.63 ± 2.88	3.44 ± 1.84	3.79 ± 1.58
AVGGC/cm	0.16 ± 0.25	0.28 ± 0.23	0.23 ± 0.36	0.2 ± 0.26
FCR	12.12 ± 0.89^b^	10.50 ± 1.01^b^	10.99 ± 0.73^b^	6.86 ± 0.20^a^
FCR_95%_CI	10.03, 14.22^b^	8.11, 12.89^b^	9.26, 12.73^b^	6.37, 7.34^a^

Values with different letters differ significantly in row (*P* < 0.05). IW, initial weight; FW, final weigh; ADG, average daily gain; AVGL, average growth in body length; AVGGH, average growth in height; AVCG, average chest growth; AVCDG, average chest depth growth; AVCWG, average chest width growth; ARHG: average recommended height growth; AVGGC, average girth circumference growth; FCR, feed conversion ratio.

### 3.2 Serum biochemical and immune indicators

Serum biochemical parameters measured after a 60-day experimental period are presented in Table 2. Dietary supplementation with varying levels of HVP had significant effect on serum levels of total cholesterol (TC) at 15d, triglyceride (TG) at 15d and 30d, and, total proteinurea (TP) at 45d and 60d in finishing goats. However, it resulted in a significant increase in total protein (TP) content in EG3 group at 60d. Results for serum immune markers are summarized in Table 3. At the study’s conclusion, interleukin-2 (IL-2) levels were significantly higher in experimental groups EG2 and EG3 (receiving HVP supplementation) compared to the control group CG1 (*p* < 0.05). In contrast, no significant differences were observed among groups for interleukin-4 (IL-4) or interleukin-6 (IL-6). Notably, based on the values of variable quantity (VQ), the levels of IL-4 and IL-6 increase as the concentration of HVP rises (comparing EG1 to EG3 versus CG1). Tumor necrosis factor-alpha (TNF-*α*) levels measured on day 60 revealed that values in the control group (CG1) were lower than those in the other three groups. During the experiment, we continuously monitored the environmental conditions to prevent excessive heat or cold. There was no occurrence of cold or heat stress in the animals. The magnitudes of the immunoglobulin and cytokine effects were all calculated precisely. Serum immunoglobulin (IgG, IgM) levels were elevated, indicating strengthened systemic immunity (comparing EG1 to EG3 versus CG1, *P* < 0.05).

**Table 2:** Serum biochemical responses in finishing goats to dietary supplementation with HVP.

Item	Date	Grouping			
		CG1	EG1	EG2	EG3
TC/(mmol/L)	0d	1.49 ± 0.22^b^	1.72 ± 0.80^a^	1.38 ± 0.27^ab^	2.26 ± 0.21^a^
	15d	1.24 ± 0.21^b^	1.19 ± 0.28^b^	1.33 ± 0.22^b^	1.85 ± 0.47^a^
	30d	1.11 ± 0.29	1.24 ± 0.34	1.22 ± 0.29	1.11 ± 0.28
	45d	1.62 ± 0.40	1.64 ± 0.37	1.55 ± 0.47	1.48 ± 0.30
	60d	1.66 ± 0.38	1.77 ± 0.61	1.32 ± 0.44	1.29 ± 0.16
	VQ	0.17	0.05	−0.06	−0.97
UN/(mmol/L)	0d	3.10 ± 0.36	3.40 ± 0.56	3.59 ± 0.59	3.25 ± 0.48
	15d	5.55 ± 1.72	4.66 ± 2.19	4.48 ± 2.00	4.60 ± 1.01
	30d	2.84 ± 0.44	3.03 ± 0.42	3.12 ± 0.23	2.79 ± 0.45
	45d	2.69 ± 0.19	3.82 ± 2.46	3.15 ± 0.19	2.84 ± 0.21
	60d	2.81 ± 0.19	2.91 ± 0.42	2.97 ± 0.28	2.73 ± 0.37
	VQ	−0.29	−0.50	−0.62	−0.52
TP/(g/L)	0d	67.33 ± 5.46	62.78 ± 7.43	61.06 ± 6.92	66.22 ± 8.55
	15d	61.63 ± 3.20	61.48 ± 9.86	59.10 ± 8.36	60.07 ± 6.46
	30d	62.60 ± 3.76	62.06 ± 7.06	63.08 ± 7.48	62.96 ± 8.67
	45d	60.30 ± 2.86^b^	62.88 ± 7.01^ab^	65.31 ± 3.66^a^	67.16 ± 4.01^a^
	60d	61.69 ± 2.73^b^	63.21 ± 6.16^b^	66.09 ± 3.74^ab^	68.61 ± 5.23^a^
	VQ	−4.33	0.08	5.03	2.40
GLU (mmol/L)	0d	4.50 ± 0.63	4,46 ± 0.51	4.55 ± 0.45	4.37 ± 0.48
	15d	4.49 ± 0.49	4.76 ± 0.81	4.65 ± 0.40	4.45 ± 0.54
	30d	4.88 ± 0.74	4.91 ± 0.35	5.24 ± 0.73	5.09 ± 0.67
	45d	5.77 ± 0.63	5.93 ± 0.59	6.08 ± 0.68	6.18 ± 0.67
	60d	5.61 ± 0.67	5.91 ± 0.59	6.00 ± 0.49	6.11 ± 0.55
	VQ	1.11	1.44	1.45	1.75
TG/(mmol/L)	0d	0.78 ± 0.52	1.08 ± 0.89	0.68 ± 0.23	1.40 ± 2.14
	15d	0.89 ± 0.29^b^	1.26 ± 0.09^ab^	1.11 ± 0.17^ab^	1.13 ± 0.22^a^
	30d	1.10 ± 0.29^a^	0.99 ± 0.15^ab^	0.86 ± 0.24^b^	0.77 ± 0.18^b^
	45d	0.94 ± 0.17	1.09 ± 0.22	0.93 ± 0.24	1.01 ± 0.26
	60d	1.00 ± 0.43	0.89 ± 0.17	1.03 ± 0.13	0.90 ± 0.33
	VQ	0.22	−0.20	0.35	−0.50

Values with different letters differ significantly in row (*P* < 0.05). TC, total cholesterol; UN, urea nitrogen; TP, total protein; GLU, glucose; TG, triglyceride; VQ, variable quantity.

**Table 3:** Serum immunological responses in finishing goats to dietary supplementation with HVP.

Item		IgA mg/ml	IgG mg/ml	IgM mg/ml	IL-2 ng/L	IL-4 ng/L	IL-6 ng/L	TNF-α ng/L
CG1	0d	2.66 ± 0.45	10.87 ± 1.29	1.89 ± 0.29	38.16 ± 5.44^ab^	67.00 ± 9.54^ab^	84.08 ± 9.85^ab^	87.82 ± 8.05
	60d	2.28 ± 0.23	7.96 ± 0.85^b^	1.82 ± 0.20^b^	30.38 ± 5.31^b^	61.38 ± 10.53	73.89 ± 8.85	75.47 ± 7.21^b^
	VQ	−0.38	−2.91	−0.07	−7.78	−5.62	−10.19	−12.35
EG 1	0d	2.88 ± 0.62	10.95 ± 1.35	2.09 ± 0.48	40.80 ± 6.08^a^	69.20 ± 9.25^a^	95.61 ± 12.94^a^	92.02 ± 10.76
	60d	2.35 ± 0.65	8.86 ± 2.39^ab^	1.72 ± 0.31^b^	31.85 ± 6.32^ab^	60.18 ± 10.40	74.62 ± 11.27	80.48 ± 13.77^ab^
	VQ	−0.53	−2.09	−0.37	−8.95	−9.02	−20.99	−11.54
EG 2	0d	2.49 ± 0.73	9.53 ± 2.37	1.87 ± 0.46	34.56 ± 5.27^b^	59.82 ± 6.82^b^	74.29 ± 15.24^b^	85.28 ± 10.44
	60d	2.69 ± 0.43	10.11 ± 1.48^a^	2.13 ± 0.23^a^	37.02 ± 4.12^a^	67.36 ± 9.45	82.35 ± 11.63	88.03 ± 6.72^a^
	VQ	0.2	0.58	0.26	2.46	7.54	8.06	2.75
EG 3	0d	2.85 ± 0.29	9.79 ± 1.31	2.09 ± 0.31	34.86 ± 4.32^b^	61.52 ± 8.95^ab^	79.25 ± 7.95^b^	85.45 ± 7.03
	60d	2.64 ± 0.50	10.16 ± 2.09^a^	2.22 ± 0.33^a^	36.23 ± 6.01^a^	69.36 ± 11.67	81.18 ± 14.22	90.78 ± 13.49^a^
	VQ	−0.21	0.37	0.13	1.37	7.84	1.93	5.33

Values with different letters differ significantly in column (*P* < 0.05). IgA, immunoglobulin A; IgG, immunoglobulin G; IgM, immunoglobulin M; IL-2, interleukin-2; IL-4, interleukin-4; IL-6, interleukin-6; TNF-α, tumor necrosis factor-alpha. VQ: variable quantity.

### 3.3 Rumen and gut microbial flora

From an initial set of 32 rumen fluid and fecal samples, six were excluded due to poor quality, leaving 26 high-quality samples for subsequent analysis (Supplementary Table S1). Quality control (QC) was performed on the raw sequencing data using the following steps: removal of adapter and barcode sequences, pair merging of reads, and quality trimming of the merged datasets. Key sequencing metrics including total read count, output yield, error rate after merging, percentages of Q20 and Q30 bases, and GC content were calculated and are detailed in Supplementary Table S1.

#### 3.3.1 OUT clustering analysis at different levels

**Figure 1:**
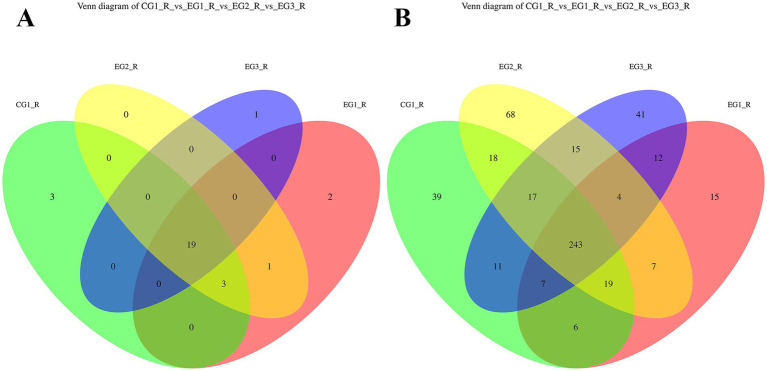
Venn diagram showing the overlap of OTUs in the gut of finishing goats supplemented with HVP (**A**: phylum level; **B**: genus level).

Based on Operational Taxonomic Unit (OTU) clustering analysis of ruminal microbiota, samples were categorized into four groups: control group CG1 (CG1_R), and experimental groups EG1 (EG1_R), EG2 (EG2_R), and EG3 (EG3_R). The corresponding Venn diagram illustrating shared and exclusive OTUs across these groups is shown in Figure 1. At the phylum level, analysis identified 29 distinct OTUs. Notably, 19 OTUs were shared among all four groups. Furthermore, CG1 possessed 3 exclusive OTUs, EG1 had 2, EG2 had none, and EG3 had 1. At the genus level, a total of 522 OTUs were detected; of these, 243 were common to all groups. Regarding group-specific OTUs, CG1 exhibited the highest richness with 39 exclusive OTUs, followed by EG2 (68), EG3 (41), and EG1 (15).

**Figure 2:**
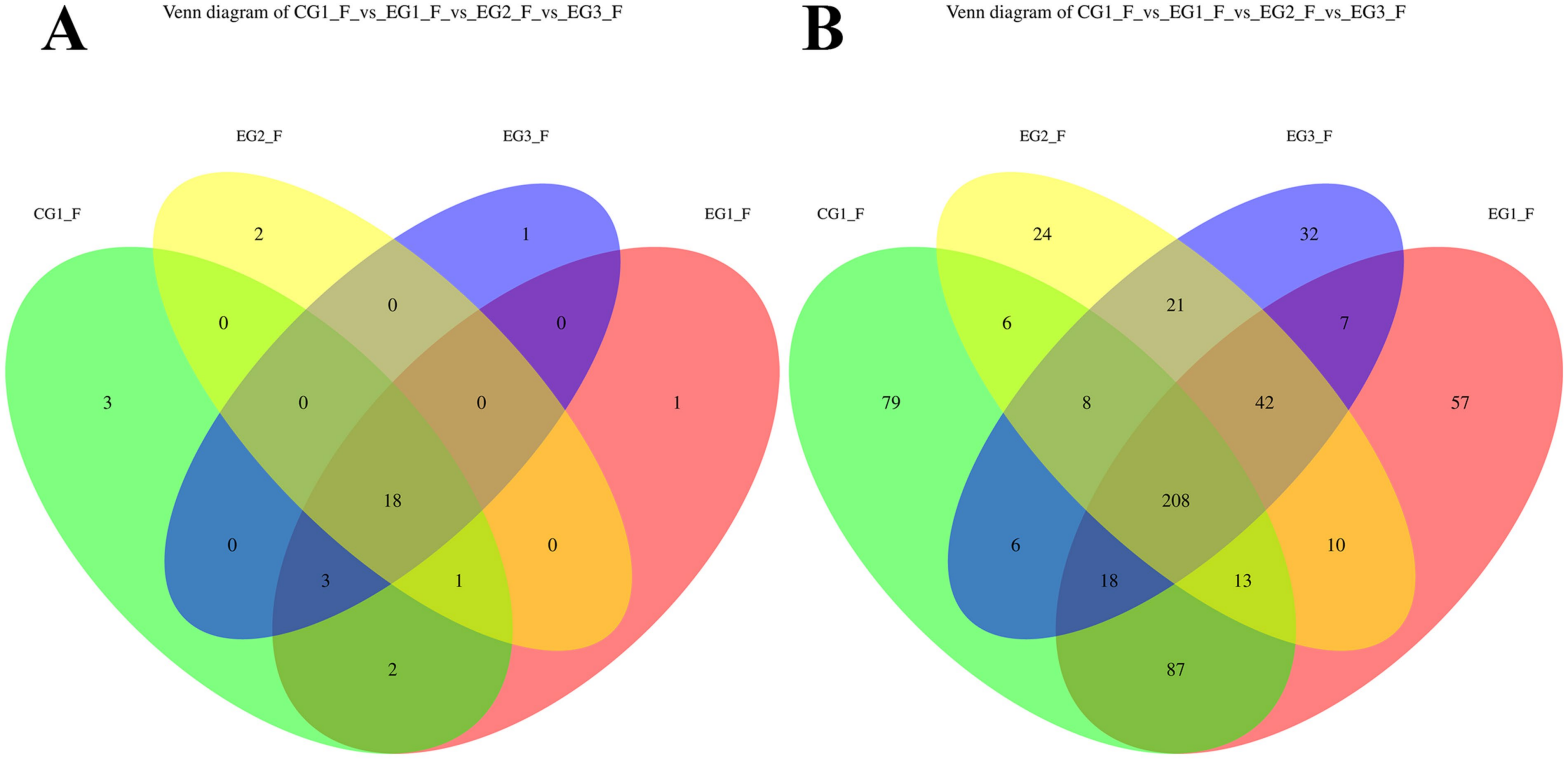
Venn diagram showing the overlap of OTUs in the gut of finishing goats supplemented with HVP (**A**: phylum level; **B**: genus level).

A parallel analysis was conducted on the gut microbiota, with groups designated as CG1 (CG1_F), EG1 (EG1_F), EG2 (EG2_F), and EG3 (EG3_F). The resulting Venn diagram is presented in Figure 2. At the phylum level, 31 OTUs were identified in total, with 18 being shared by all four groups. In terms of exclusivity, CG1 contained 3 unique OTUs, EG1 had 1, EG2 had 2, and EG3 had 1. At the genus level, from a total of 618 clustered OTUs, 208 were shared across all groups. For group-specific OTUs, CG1 showed the greatest abundance with 79 exclusive OTUs, while the treatment groups had 57 (EG1), 24 (EG2), and 32 (EG3), respectively.

#### 3.3.2 Analysis of microbial alpha diversity

**Figure 3:**
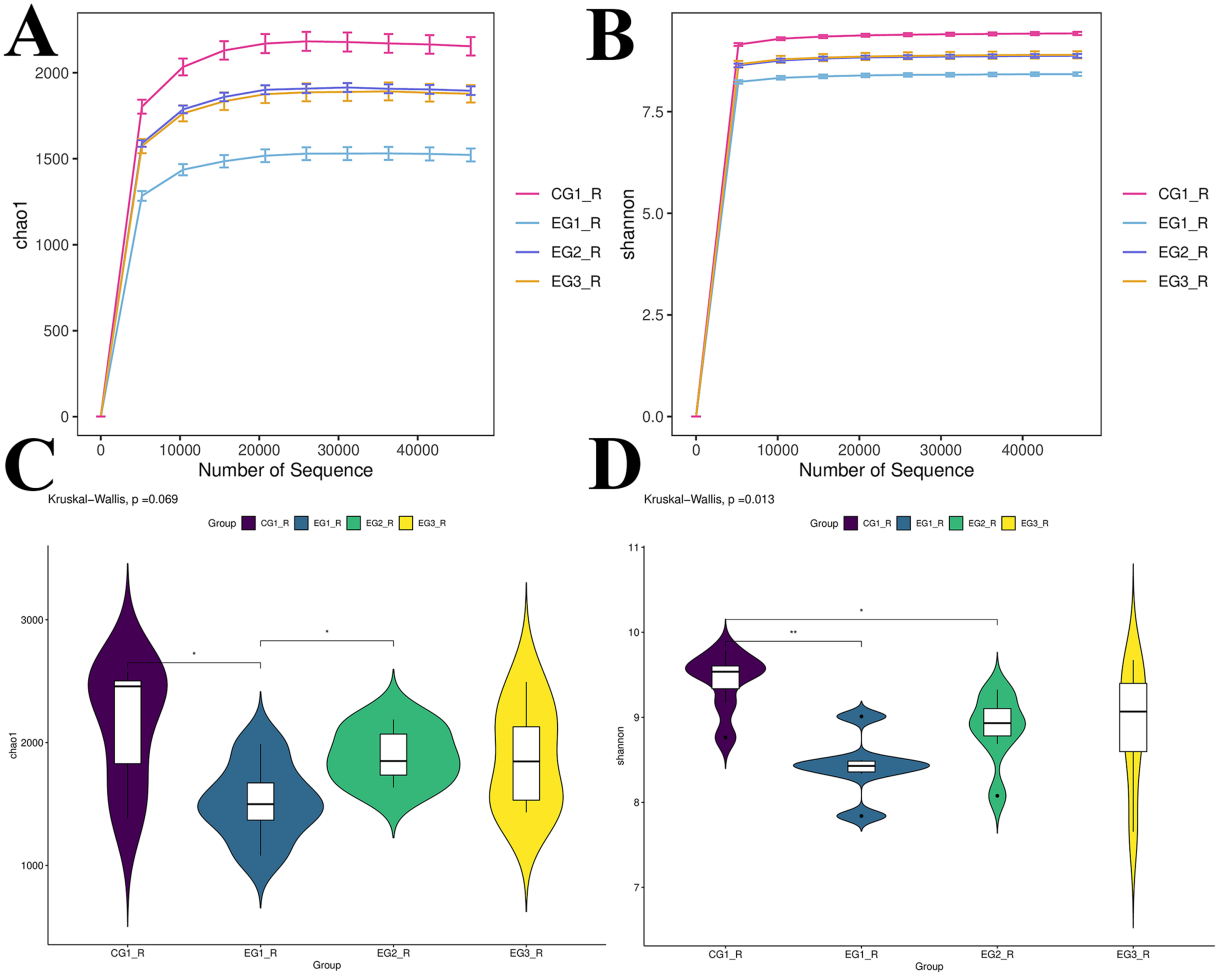
Alterations in ruminal microbial alpha diversity following HVP supplementation in fattening goats. * Indicates significant difference (*p* < 0.05). **(A)** Represents the Chao1 index; **(C)** represents the Shannon index; **(B,D)** compares and analyzes the Chao1 index and Shannon index of the rumen microbiota of four groups of fattening goats.

High-throughput sequencing targeted at the V3 and V4 hypervariable regions of the 16S rRNA gene was performed on all experimental samples using universal primers. Rarefaction curves for both richness and incidence demonstrated that species accumulation progressively plateaued, indicating sufficient sequencing depth to capture the majority of rumen microbial taxa (Figures 3A,C). To compare alpha diversity among groups, we employed ANOVA on commonly used metrics: the Chao1 estimator of species richness and the Shannon index, which accounts for both richness and evenness. Significant differences were observed in the Chao1 index between the control group (CG1) and experimental group 1 (EG1; *p* < 0.05), as well as between EG1 and EG2 (*p* < 0.05). For the Shannon index, significant differences were detected between CG1 and EG1 (*p* < 0.01) and between CG1 and EG2 (*p* < 0.05). These results revealed distinct responses of different diversity facets to the experimental treatments. Notably, both estimated richness (Chao1) and overall diversity (Shannon) decreased in certain treatment groups relative to the control group (Figures 3B,D).

**Figure 4:**
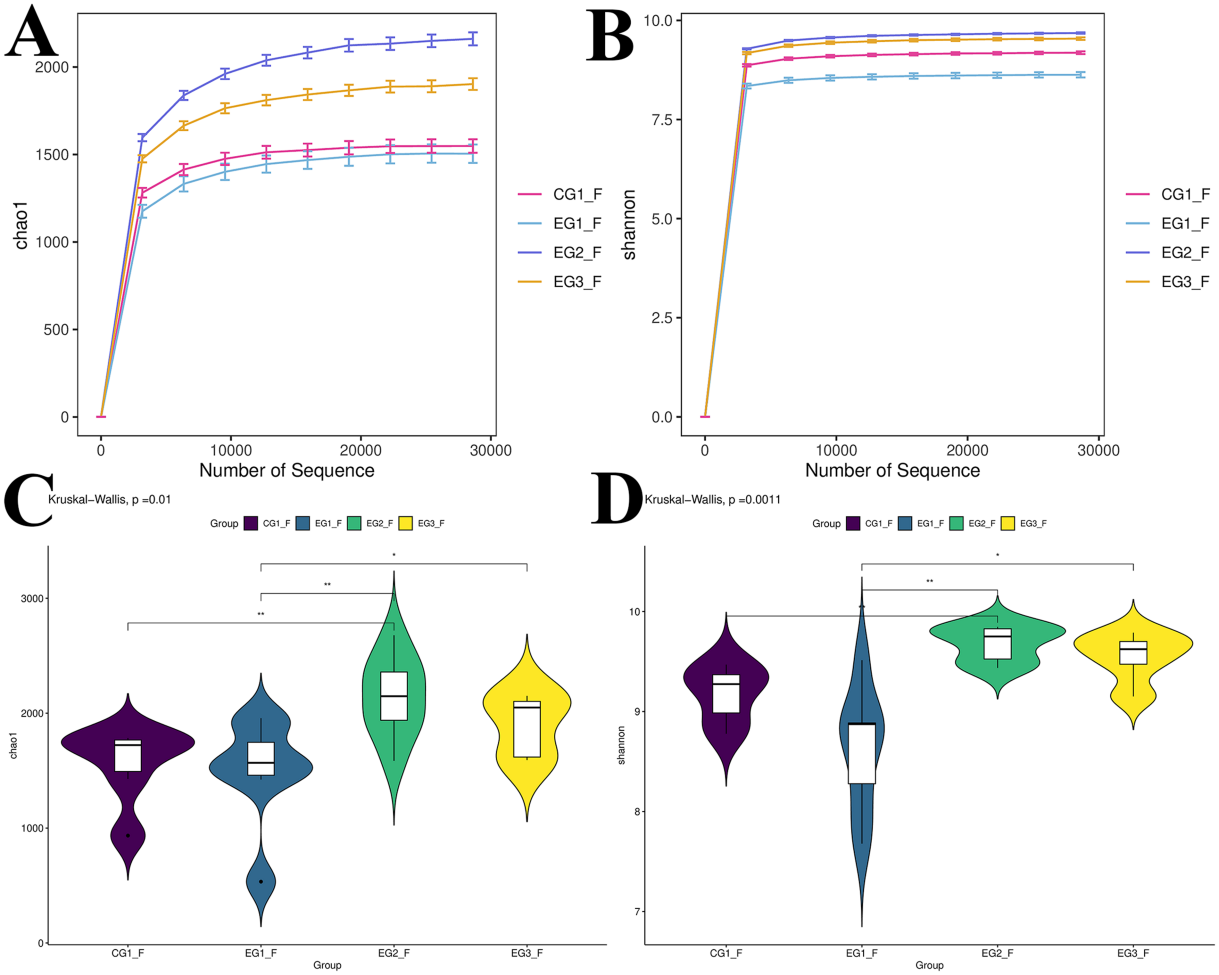
Alterations in gut microbial alpha diversity following HVP supplementation in fattening goats. * Indicates significant difference (*p* < 0.05). **(A)** Represents the Chao1 index; **(C)** represents the Shannon index; **(B,D)** compares and analyzes the Chao1 index and Shannon index of the intestinal microbiota of four groups of fattening goats.

Shannon diversity and Chao1 richness curves were constructed from the microbial diversity data of individual fecal samples across varying sequencing depths, as illustrated in Figures 4A,C. With an increasing number of sequences reads, the estimated species richness of the goat gut microbiota correspondingly increased. When these curves approached a plateau, it signified that the volume of sequencing data was adequate to represent the main microbial communities within the samples. According to the sequencing analysis, a significant difference in the Chao1 index was observed between the EG2 group and the control group (CG1; *p* < 0.01), and between the EG1 and EG2 groups (*p* < 0.01). Additionally, a significant difference was found between the EG3 and EG1 groups (*p* < 0.05). Regarding the Shannon index, there was a significant difference between the EG2 group and the CG1 group (*p* < 0.01). The EG1 group possessed a significantly lower Shannon index than the EG2 group (*p* < 0.01). Moreover, a significant difference was detected between the EG3 and EG1 groups (*p* < 0.05) (Figures 4B,D).

#### 3.3.3 Differences in rumen microbial community composition and abundance

**Figure 5:**
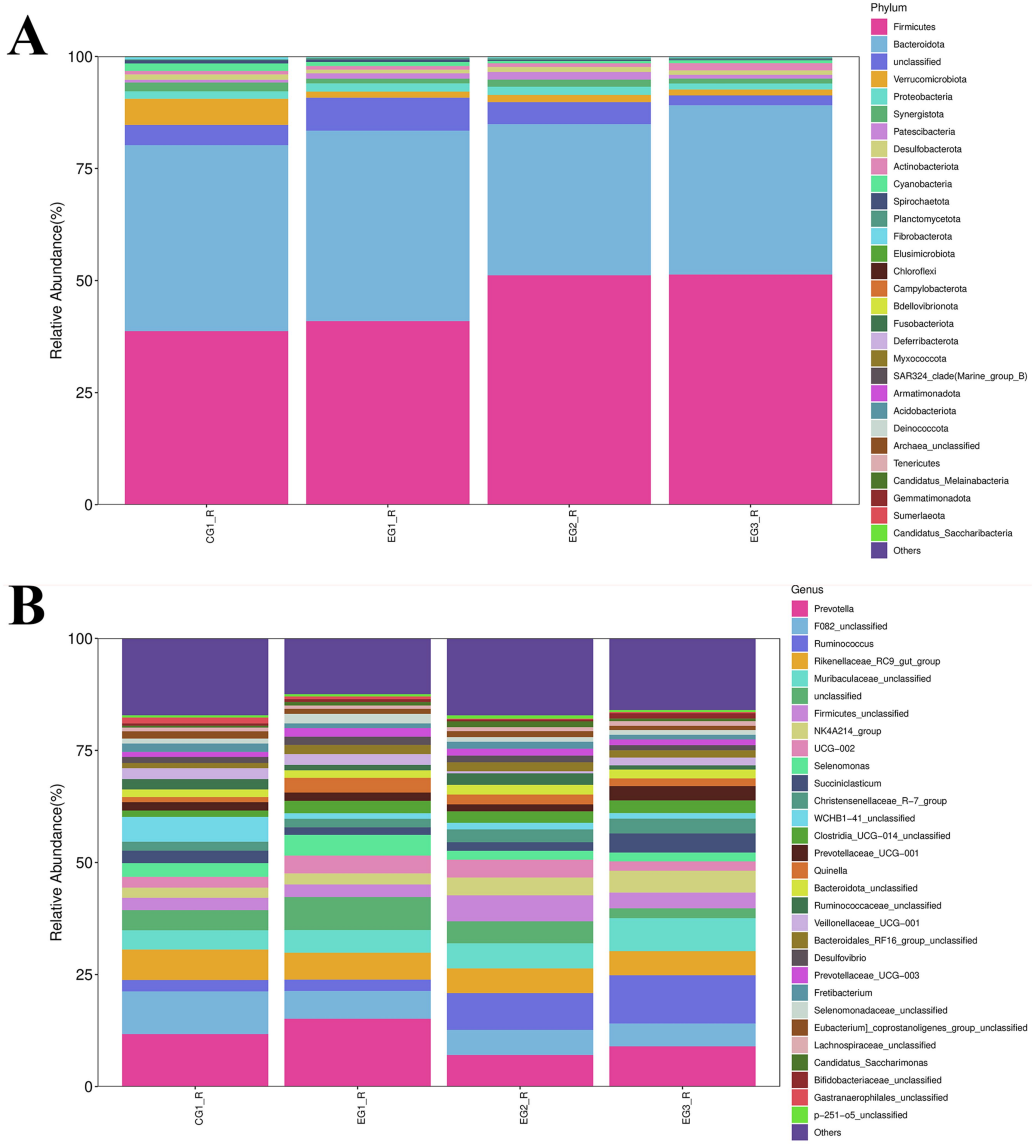
Impact of HVP on rumen microbiota composition in goats **(A)** phylum level; **(B)** genus level.

As shown in Figure 5A, analysis of the rumen microbiota composition in goats revealed that, at the phylum level, Firmicutes dominated across all four groups (CG1_R, EG1_R, EG2_R, and EG3_R), with relative abundances of 38.72, 40.96, 51.19, and 51.36%, respectively. Concurrently, Bacteroidota represented the second most abundant phylum in these same groups, accounting for 41.51, 42.47, 33.71, and 37.74% of the sequences, respectively. Together, these two phyla consistently comprised over 80% of the community in each group, establishing them as the dominant components of the rumen microbiome. Furthermore, the relative abundance of Verrucomicrobiota varied significantly among groups: it was highest in CG1_R (5.84%) compared to much lower levels observed in EG1_R (1.35%), EG2_R (1.59%), and EG3_R (1.31%). For Proteobacteria, relative abundances were 1.65, 1.83, 1.84, and 1.35% in CG1_R, EG1_R, EG2_R, and EG3_R, respectively. Similarly, Synergistota exhibited low but detectable abundances of 1.96, 1.05, 1.59, and 1.11% across the four groups.

As illustrated in Figure 5B, analysis at the genus level (excluding operational taxonomic units classified as “Others”) revealed that Prevotella was highly abundant across all four groups, consistently exceeding 5% relative abundance. Specifically, its proportions were 11.72, 15.14, 7.07, and 8.99% in CG1_R, EG1_R, EG2_R, and EG3_R, respectively. Among other prominent genera, F082_unclassified displayed abundances of 9.53, 6.19, 5.59, and 5.09%, while members of the Rikenellaceae_RC9_gut_group constituted 6.80, 5.99, 5.51, and 5.37% of the respective communities. Notably, several genera increased in abundance correlating with higher proportions of HVP (highly variable sequences? assuming HVP definition is known): these included Ruminococcus (2.54% in CG1_R, 2.54% in EG1_R, 8.20% in EG2_R, and 10.77% in EG3_R), Muribaculum (or Muribaculaceae_unclassified; 4.28, 5.08, 5.59, and 7.35%), and WCHB1-41_unclassified (5.53, 1.22, 1.48, and 1.24%).

**Figure 6:**
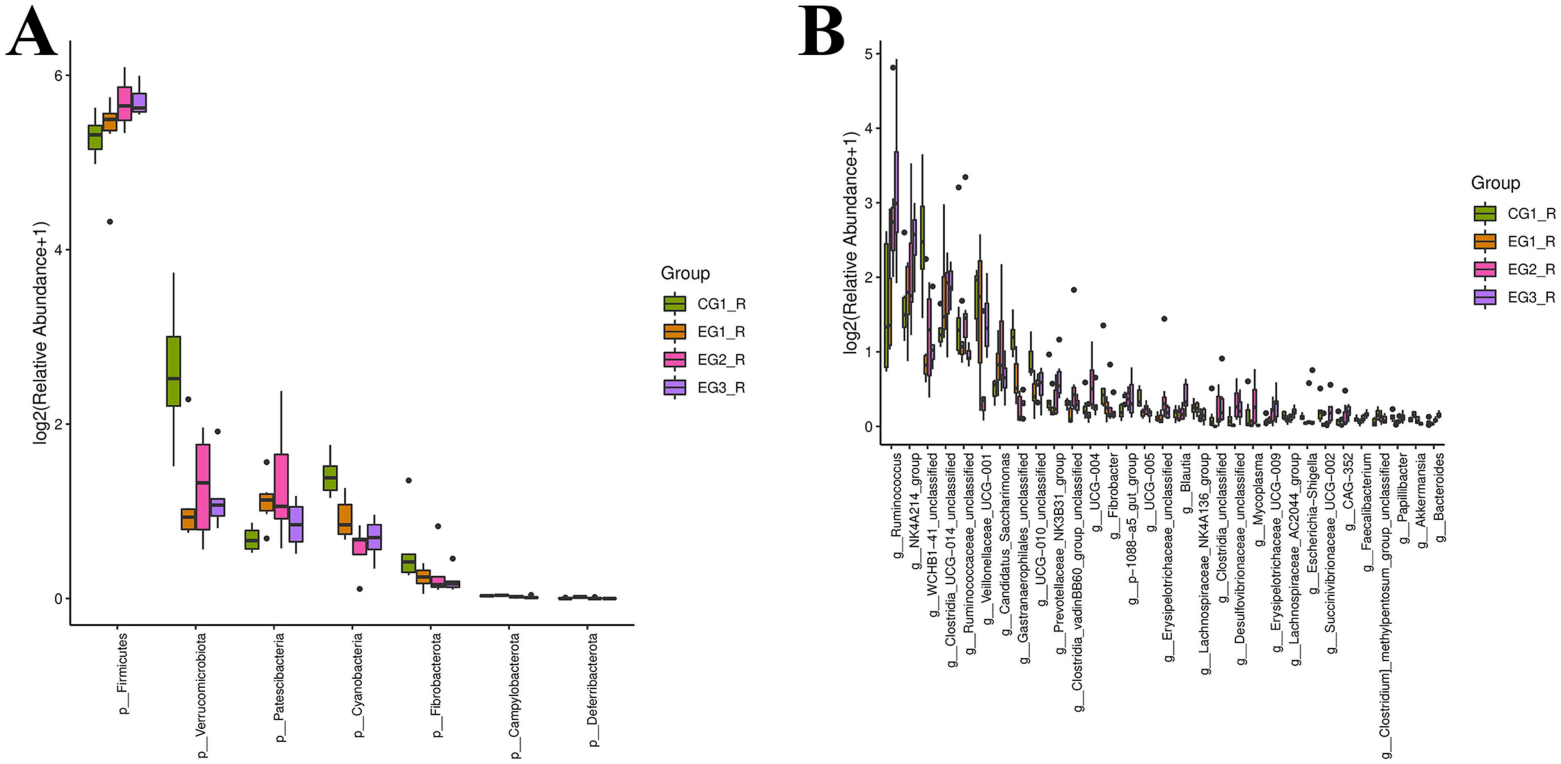
Taxonomic profiling and community structure of rumen microbiota in goats **(A)** phylum level; **(B)** genus level.

To investigate shifts in the rumen microbiota composition more thoroughly, we conducted differential abundance analyses at both phylum and genus taxonomic levels (Figure 6). At the phylum level, seven bacterial phyla showed statistically significant differences among groups. Specifically, within the control group (CG1), Verrucomicrobiota, Cyanobacteria, and Fibrobacterota exhibited significantly higher relative abundances compared to the three experimental groups. Conversely, Patescibacteria displayed a significantly lower abundance in CG1. In contrast, the EG2_R group demonstrated significantly elevated abundances of both Firmicutes and Patescibacteria relative to the other two groups, whereas its abundance of Cyanobacteria was significantly diminished. At the genus level, analysis identified 63 taxa with significant differential abundance. Among these, g__WCHB1-41_unclassified and g__GastranaeroPhilales_unclassified attained their peak abundances in the CG1_R group. The highest abundance of g__Veillonellaceae_UCG-001 was observed in the EG1_R group. Meanwhile, g__Ruminococcus, g__NK4A214_group, and g__Clostridia_UCG-014_unclassified were most abundant in the EG3_R group.

#### 3.3.4 Characterizing differences in gut microbiota composition and diversity

**Figure 7:**
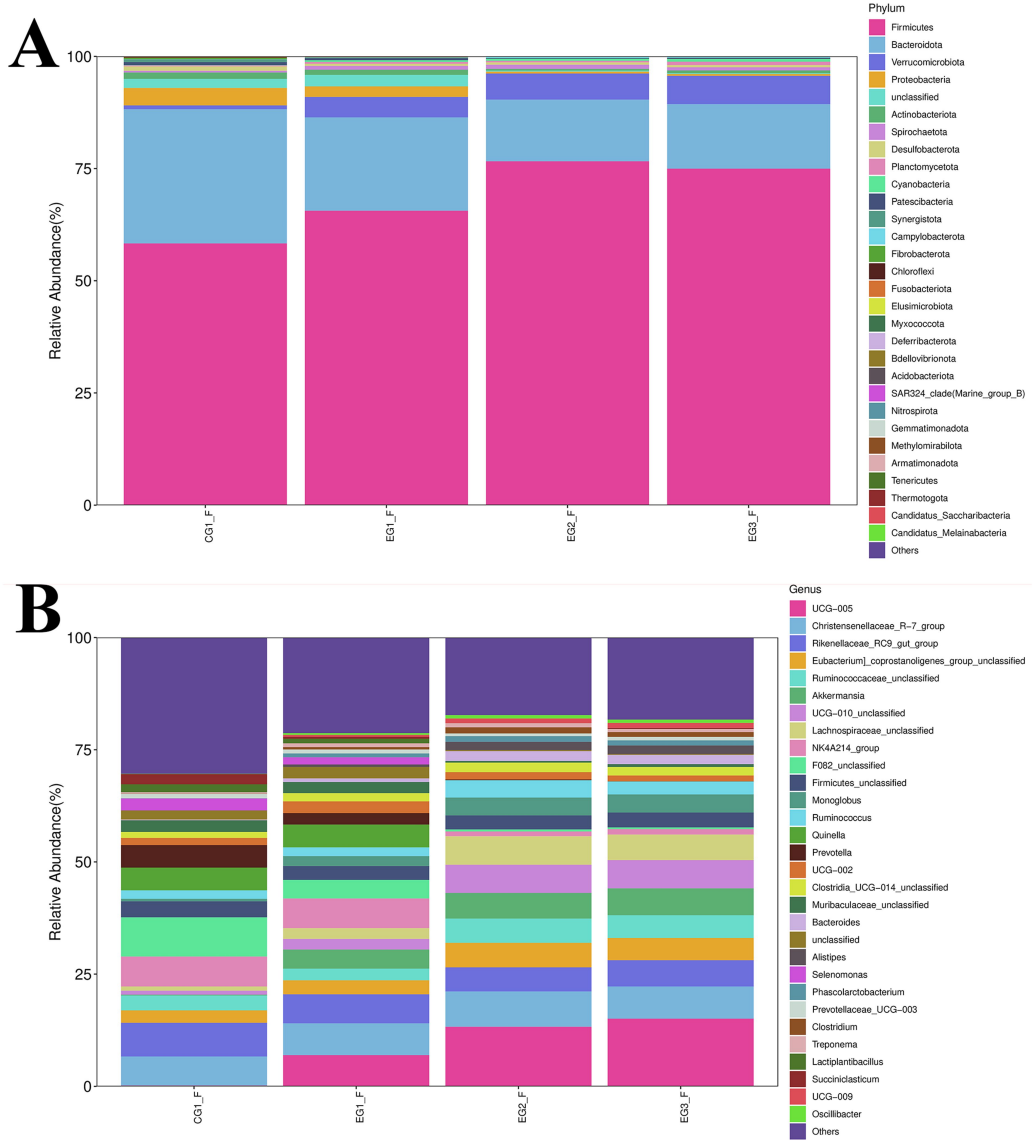
Impact of HVP on gut microbiota composition in goats **(A)** phylum-level taxonomic distribution; **(B)** genus-level taxonomic resolution.

As shown in Figure 7A, which displays the intestinal microbiota profiles of finished goats at the phylum level, Firmicutes and Bacteroidota were the most abundant phyla in the control group, with relative abundances of 58.32 and 29.91%, respectively, followed by Proteobacteria at 3.90%. In contrast, within the experimental groups (EG1_F, EG2_F, EG3_F), the dominant phyla shifted to include Verrucomicrobiota alongside Firmicutes and Bacteroidota. Specifically, their abundance distribution was as follows: for EG1_F – Firmicutes (65.63%), Bacteroidota (20.80%), Verrucomicrobiota (4.53%); for EG2_F – Firmicutes (76.62%), Bacteroidota (13.76%), Verrucomicrobiota (5.80%); and for EG3_F – Firmicutes (74.98%), Bacteroidota (14.38%), Verrucomicrobiota (6.32%). Notably, compared to the CG1 group, the experimental groups exhibited an increase in the relative abundance of both Bacteroidota and Proteobacteria, while a decrease was observed in Firmicutes.

At the phylum level, experimental groups showed increased abundances of both Firmicutes and Verrucomicrobiota. As illustrated in Figure 7B (genus-level analysis, excluding “Others”), the CG1 group displayed notably higher proportions of Christensenellaceae_R-7_group (6.46%), Rikenellaceae_RC9_gut_group (7.50%), NK4A214_group (6.67%), and Prevotella (5.01%). In contrast, experimental groups were characterized by elevated levels of UCG-005, Christensenellaceae_R-7_group, Rikenellaceae_RC9_gut_group, and Akkermansia. Specifically: EG1_F: 6.95% (UCG-005), 7.10% (Christensenellaceae_R-7_group), 6.47% (Rikenellaceae_RC9_gut_group), 4.26% (Akkermansia) EG2_F: 13.29% (UCG-005), 7.86% (Christensenellaceae_R-7_group), 5.37% (Rikenellaceae_RC9_gut_group), 5.70% (Akkermansia) EG3_F: 15.08% (UCG-005), 7.16% (Christensenellaceae_R-7_group), 5.87% (Rikenellaceae_RC9_gut_group), 6.00% (Akkermansia) Notably, compared to the CG1 group, all three experimental groups demonstrated significantly higher relative abundances of Rikenellaceae_RC9_gut_group, NK4A214_group, and Prevotella. Conversely, the CG1 group exhibited lower abundances of Christensenellaceae_R-7_group and UCG-005 than those observed in the experimental cohorts.

**Figure 8:**
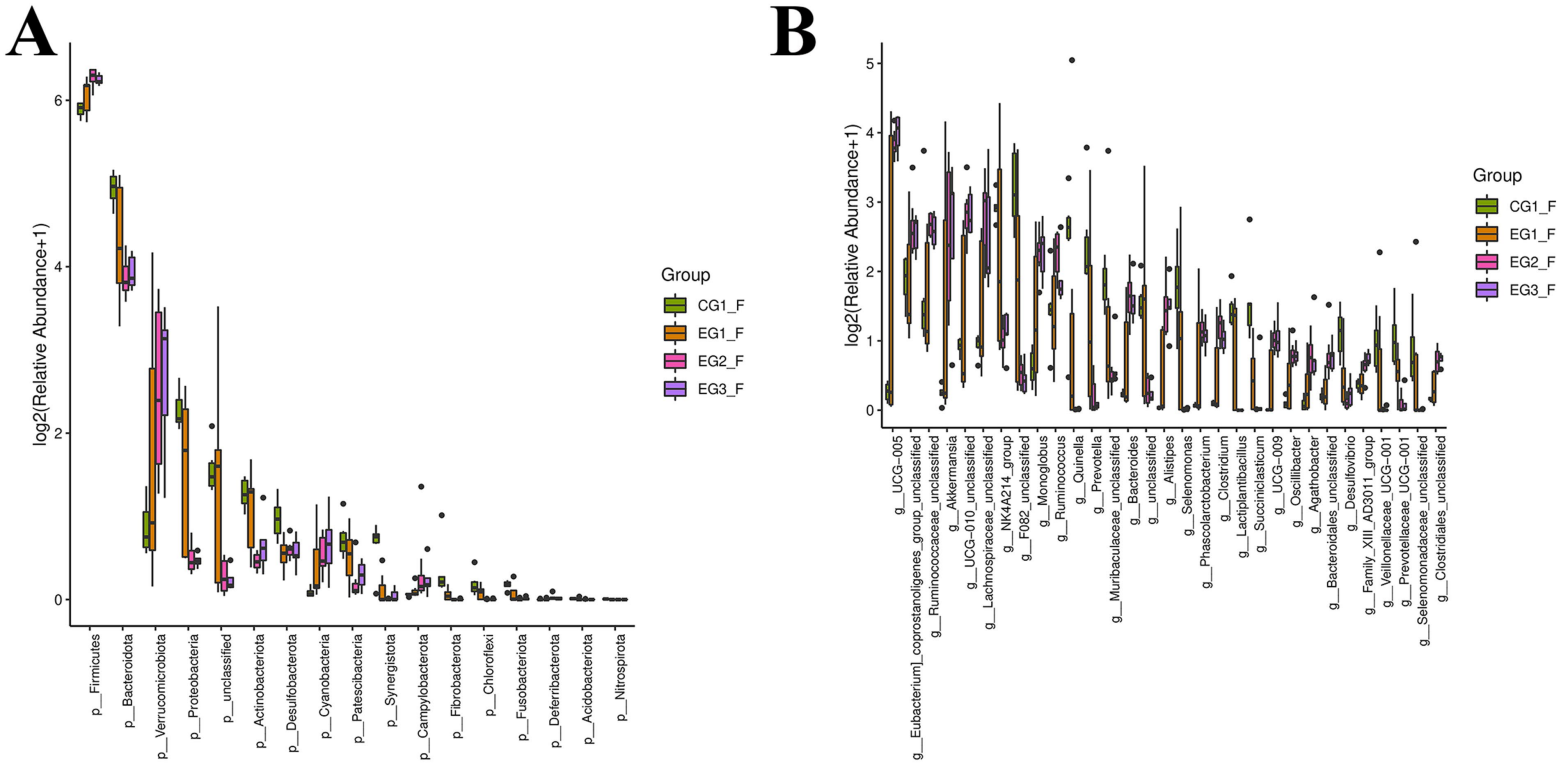
Community assembly patterns of gut microbiota in goats exposed to HVP **(A)** phylum-level abundance profiles; **(B)** genus-level operational taxonomic units.

Figure 8A presents a differential analysis of gut microbiota composition at the phylum level, revealing significant intergroup variations among 17 bacterial phyla. Compared to the other three groups, the CG1 group exhibited significantly lower abundances of P__Firmicutes and P__Verrucomicrobiota. In contrast, this group displayed markedly higher levels of P__Bacteroidota, P__Proteobacteria, and P__Actinobacteriota. Notably, both EG2_F and EG3_F groups showed reduced abundances of P__Bacteroidota, P__Proteobacteria, P__unclassified, and P__Actinobacteriota, coupled with elevated proportions of P__Verrucomicrobiota and P__Cyanobacteria.

At the genus level (Figure 8B), 217 bacterial taxa displayed significant abundance differences across groups. Relative to the CG1 group, the EG2_F and EG3_F cohorts had increased abundances of g__UCG-005, g__Eubacterium_coprostanoligenes_group_unclassified, g__Ruminococcaceae_unclassified, g__Akkermansia, g__UCG-010_unclassified, and g__Lachnospiraceae_unclassified. Conversely, these experimental groups demonstrated decreased abundances of NK4A214_group, F082_unclassified, Prevotella, and Muribaculaceae_unclassified.

## 4 Discussion

### 4.1 Supplementation with HVP improved growth performance and boosted immune function in Nanjiang Yellow goat during the finishing period

Honeysuckle is rich in various organic acids and polyphenols. These bioactive constituents may promote the secretion of gastric juices, increase digestive enzyme activity, stimulate gastrointestinal motility, and facilitate lipid digestion. Such effects can lead to enhanced appetite and feed intake in young animals, ultimately supporting healthy weight gain. Supporting this potential, Zhang et al. reported that dietary supplementation with a Chinese herbal mixture containing honeysuckle improved growth performance, immunity, antioxidant status, and gut microbiota composition in calves (Zhang et al., 2025). Further evidence highlights the role of honeysuckle’s key component, chlorogenic acid (CGA).

In this study, supplementing finishing goat diets with HVP significantly improved their growth performance. Key metrics including net weight gain, average daily gain (ADG), and feed conversion ratio (FCR) were markedly enhanced following HVP supplementation. Notably, considerable intergroup variation in FCR was observed. We hypothesize that this may be attributable to the transition from the primary grazing regimen typical of Nanjiang Yellow goats to a stall-feeding system, which likely induced initial stress responses across all groups. However, as the dietary inclusion rate of HVP increased, corresponding improvements in FCR were noted. This suggests that HVP may possess adapt genic properties helping to mitigate stress in newly confined animals. Our findings resonate with those of Ma et al. reported increased dry matter intake in heat-stressed dairy cows receiving dietary honeysuckle extract (Ma et al., 2020).

Serum biochemical parameters provide objective insights into animal health, metabolic status, and overall welfare (Galapero et al., 2015). Blood urea nitrogen (BUN), a key indicator of dietary protein utilization and amino acid homeostasis (Stanley et al., 2002), remained unaffected by experimental treatments throughout this study. At the 15-day mark, there was a certain growth trend in BUN in CG1, EG1, and EG2 (Table 2). This transient elevation is likely attributable to dual stressors: the transition from pasture grazing to confinement feeding and an abrupt temperature drop during the autumn-winter shift in the mountainous region. Such environmental pressures may have induced physiological strain, potentially exacerbated by susceptibility to cold-related ailments. Following recovery from cold exposure, all groups returned to baseline BUN levels, with no significant intergroup or temporal differences observed thereafter. These findings collectively indicate that supplementation with honeysuckle vine powder (HVP) did not adversely impact protein metabolism or renal function in Nanjiang Yellow goats.

Total protein (TP) serves as a critical marker for assessing metabolic activity and immune competence (Vicari et al., 2008). While initial TP variations existed across groups, they lacked statistical significance. By day 15, TP declined universally, though the magnitude was smallest in group EG1—a trend possibly linked to concomitant increases in BUN during the adaptation period. Subsequently, at day 45, experimental groups showed elevated TP levels with significant divergence between EG2, EG3, and CG1. By day 60, EG3 exhibited distinct separation from other groups (vs. EG1 and CG1). Serum glucose (GLU), reflective of systemic energy metabolism, shows a positive correlation with the feed conversion efficiency, but no significant intergroup differences were detected at any timepoint (Table 2). Similarly, while triglycerides (TG)—the principal storage form of lipid energy (Alves-Bezerra and Cohen, 2018)—showed no group differences initially (day 0) or finally (day 60), marked fluctuations occurred between days 15–30. This pattern mirrored BUN dynamics during the same window. As dietary energy density rose, individual variations in TG accumulation emerged; hosts subsequently mobilized stored fat reserves to meet energetic demands. A discernible reduction in TG accompanied HVP supplementation, presumably due to bioactive phytochemicals within honeysuckle by-products (Han et al., 2016; Li et al., 2020). This effect is similar to the reduction in triglycerides observed after chronic intravenous administration of chlorogenic acid in rats (de Sotillo and Hadley, 2002) and after dietary inclusion of 1% honeysuckle leaf powder in finishing pigs (Qin-hua et al., 2016).

Serum immunoglobulin levels constitute a key index of systemic immune competence, as these antibodies are fundamental to protecting animals from pathogenic invasion (Luo et al., 2021; Ma et al., 2022). At baseline (day 0), no significant intergroup differences were observed in IgA, IgG, or IgM concentrations. However, by day 60, both the EG2 and EG3 groups exhibited significantly elevated IgM levels compared to controls, indicating that HVP supplementation effectively enhances immunoglobulin production. This effect may stem from the high flavonoid content in honeysuckle by-products like vine leaves (Li et al., 2020), compounds renowned for their potent antibacterial properties. Upon entering the host, these bioactive molecules can fortify immune cell function and amplify cell-mediated immunity, enabling more efficient clearance of viral threats and thereby improving overall immunosurveillance. Furthermore, chlorogenic acid—a major active constituent within honeysuckle matrixes—has been shown to suppress pro-inflammatory cytokine expression (Park et al., 2015). Tumor necrosis factor alpha (TNF-*α*), a pleiotropic mediator central to immune signaling, orchestrates defensive cascades by activating macrophages and stimulating downstream production of regulatory mediators (Zhang et al., 2003; Koss et al., 2006). Consistent with prior reports, our data demonstrate that incremental increases in dietary HVP correspond to dose-dependent rises in circulating TNF-α concentrations. As a ubiquitous immune modulator, TNF-α occupies a critical node in the body’s defense network.

Interleukin-2 (IL-2) drives proliferation and differentiation of T lymphocytes, B lymphocytes, and natural killer (NK) cells while promoting secretion of secondary cytokines including interferons and additional TNF isoforms. Through these mechanisms, IL-2 bolsters host resistance against disease (Malek, 2003). Concurrently, IL-4 stimulates growth and expansion of B cell populations, directly facilitating immunoglobulin class switching and follicular hyperplasia to support humoral immunity. The multifunctional cytokine IL-6 plays indispensable roles in B lymphocyte maturation and germination center formation. It also directs IgG secretion to maintain endogenous homeostasis. Upon detection of exogenous pathogens, phagocytes release IL-6 into systemic circulation; this signal traffics to the liver where it triggers synthesis of mannose-binding lectin, thereby launching multi-tiered innate immune responses (Janeway, 1993; Diehl et al., 2000; Smith et al., 2013). Notably, we observed dynamic modulation of IL-2 expression across treatment groups. We postulate that chronic dietary administration of HVP helps preserve immunological order by sustaining elevated immunoglobulin secretion and macrophage activation—collectively enhancing adaptive and innate arms of host defense. Compared to controls (CG1 group), both IL-4 and IL-6 were upregulated following HVP intervention, suggesting dual mechanisms: augmented B cell effector functions alongside constrained pathological inflammation during practical application scenarios.

### 4.2 Incorporating HVP into the diet modulates rumen and gut microbial communities in Nanjing Yellow goats

As the central digestive organ in ruminants, the rumen harbors a complex and dynamic microbial ecosystem comprising bacteria, protozoa, and fungi that collectively sustain host digestion (Qu et al., 2023). The composition of this microbiota is primarily shaped by dietary inputs. Analysis of alpha diversity metrics, Chao1 and Shannon indices revealed significant discrepancies between the experimental group and control group (CG1), indicative of differences in species richness and evenness. Dietary supplementation with distinct feed additives is known to modulate the gastrointestinal microbiome of livestock, subsequently influencing ruminant metabolism (Liu et al., 2021). In our study, Firmicutes and Bacteroidota emerged as the dominant bacterial phyla within the rumen, a finding consistent with established profiles of ruminant ruminal microbiota (Singh et al., 2012; Shen et al., 2017). Supplementation with varying proportions of HVP resulted in a dose-dependent increase in the relative abundance of Firmicutes at the phylum level, rising from 38.72% in CG1 to 51.36% in the group receiving 2% HVP inclusion. Firmicutes, renowned for their fibrolytic capabilities, displayed an altered ratio relative to Bacteroidota following HVP supplementation. Specifically, the experimental groups exhibited higher Firmicutes abundance coupled with lower Bacteroidota levels, a pattern corroborated by prior research on dairy cow nutrition involving rumen contents and feed composition (Jami et al., 2014). Accumulating evidence (Ley et al., 2006; Turnbaugh et al., 2006; Jami et al., 2014) links the Firmicutes: Bacteroidota ratio to critical production traits such as milk yield, meat quality, and physiological performance in livestock. Furthermore, studies in humans and mice demonstrate that an elevated Firmicutes: Bacteroidota ratio is strongly associated with increased body fat accretion; obese individuals typically exhibit higher Firmicutes and lower Bacteroidota abundance (Qadir and Assafi, 2021). Based on these premises, we infer that HVP supplementation enhances fermentable fiber degradation within the rumen. This conclusion aligns with our earlier observation of increased body weight gain correlating with higher HVP inclusion rates reported in the production performance section.

Verrucomicrobiota, implicated in energy-intensive processes like cellular transport and host metabolism/growth (Li et al., 2016), typically declines under high-fiber diets. We hypothesize that HVP addition may reduce effective fiber intake, thereby explaining the lower Verrucomicrobiota abundance observed in experimental groups compared to controls (CG1). At the genus level, Prevotella plays a pivotal role in host digestion, possessing enzymes to degrade proteins, peptides, and polysaccharides. Its abundance positively correlates with propionate production, a short-chain fatty acid crucial for energy metabolism. Conversely, increased abundance of Rikenellaceae has been associated with reduced visceral fat accumulation, promoting a healthier metabolic profile in hosts (Precup and Vodnar, 2019; Tavella et al., 2021). Our results identified Prevotella, Ruminococcus, and Muribaculaceae as key responsive genera. Notably, Prevotella abundance decreased with increasing HVP dosage, while Ruminococcus and Muribaculaceae showed opposing trends (increased abundance). Since both Rikenellaceae and Prevotella can influence fat deposition rates, their dynamic abundance necessitates careful management within target ranges during production to optimize growth rates. As a primary fiber-degrader, Ruminococcus abundance increased significantly post-HVP supplementation, indicating enhanced colonization by this functionally important bacterial group. Previous work (Kong et al., 2016) identified Ruminococcus as a potential probiotic due to its roles in shaping the intestinal environment, modulating inflammation and cytotoxicity, supporting immune function, and maintaining homeostasis. This finding resonates with our earlier observations linking HVP supplementation to stabilized rumen immunity via elevated Ruminococcus levels (Wang et al., 2021). Supportingly, murine studies (Tong et al., 2018) show positive correlations between Muribaculaceae abundance and key dairy traits, such as milk yield, fat percentage, protein content, and feed efficiency. Crucially, in practical settings, increases in both Ruminococcus and Muribaculaceae occur simultaneously (Jiang et al., 2020).

Multiple factors, such as feed additive types, dietary formulation, genetic traits, heat stress exposure, and management practices like feeding strategies can profoundly shape the gut microbial ecosystem and overall health of livestock (Nawab et al., 2018). Functioning as a dynamic interface, the gut harbors a unique microbial community and metabolome that work in concert to maintain microbial balance and positively influence host fitness (Kuziel and Rakoff-Nahoum, 2022). In this study, supplementation with honeysuckle vine powder (HVP) enhanced both the diversity and richness of the gut microbiota in Nanjiang Yellow goats, strengthened their gut immunity, and supported a stable microbial equilibrium. These findings resonate with prior observations on HVP’s effects on the gut microbiota of Hu sheep (Li D. et al., 2023; Li Y. et al., 2023).

At the phylum level, Firmicutes, renowned for their high efficacy in energy harvest, are critically involved in host energy acquisition and immune regulation. An increased abundance of this group is often linked to meeting the elevated energy demands associated with muscle growth (Du et al., 2022). Members of the Firmicutes possess the metabolic capacity to extract calories from ingested nutrients, fueling essential physiological processes. Consistent with this role, we observed that increasing HVP inclusion levels correlated with higher Firmicutes abundance in our experimental groups, which coincided with improved growth performance in goats. Conversely, Bacteroidota play a key role in digesting complex carbohydrates, breaking down non-fibrous polysaccharides, and preserving gut homeostasis. Notably, studies in humans have established an association between obesity and a lower Bacteroidota-to-Firmicutes ratio; weight loss interventions typically lead to an increase in Bacteroidota and a decrease in Firmicutes (Hales et al., 2017; Jie et al., 2021). Our data mirrored this pattern, showing a positive correlation between body weight ratio and the Firmicutes/Bacteroidota ratio across different treatment groups. Given their prevalence and functional significance, this ratio emerges as a potential biomarker for monitoring gut dysbiosis.

Furthermore, the abundance of Verrucomicrobiota increased progressively with higher HVP supplementation levels among the four experimental groups. Previous research has documented that broad-spectrum antibiotic treatment elevates Verrucomicrobiota levels in the human gut (Dubourg et al., 2013). Based on this observation and our own findings, we hypothesize that the proliferation of Verrucomicrobiota due to HVP supplementation may contribute to enhanced disease resistance in goats.

Moving to the genus level, UCG-005 was a member of the Ruminococcaceae family, exhibited a significant difference in relative abundance between the control group and the experimental groups. As a dominant taxon in ruminant rumens, UCG-005 is instrumental in degrading cellulose and other polysaccharides, thereby benefiting host gut health (Zhi et al., 2022). This genus is known for its prolific production of cellulases and hemicellulases, enabling the conversion of dietary fiber into essential nutrients for the host and playing a central role in ruminant digestion and carbohydrate metabolism (La Reau and Suen, 2018). Another notable genus, Christensenellaceae_R-7_group, is primarily engaged in amino acid and lipid metabolism. Considered potentially beneficial, these bacteria contribute to a favorable gut environment and are implicated in immune modulation and healthy homeostasis, likely resulting from long-term coevolution with their hosts (Zhang et al., 2022). In our experiment, both UCG-005 and Christensenellaceae_R-7_group showed increased abundance in the experimental groups. We postulate that these dominant bacterial genera help goats more effectively degrade cellulose, improve nutrient absorption efficiency, enhance immune function, better adapt to indoor housing conditions, and ultimately boost their productive performance.

Given the results of this study, we suggest that future research could explore precision nutrition. To enhance the potential for improving the growth performance of ruminants, analyses of rumen fermentation, macrogenomics/macrotranscriptomics, and metabolomics will be conducted. These experiments will provide us with a more comprehensive perspective to understand how nutritional supplements affect the physiological and metabolic processes of ruminants. Through these studies, we can better design precision nutrition plans to enhance the production efficiency and health levels of ruminants.

## 5 Conclusion

In conclusion, our experimental findings demonstrate that dietary supplementation with 2% HVP may enhance growth performance and feed efficiency in Nanjiang Yellow goats, as evidenced by improved daily weight gain and feed conversion ratio. Concomitant increases in serum IgG and IgM levels indicate a bolstered humoral immune response, suggesting that HVP exerts immunomodulatory effects in this ruminant species. Notably, HVP supplementation led to a marked shift in ruminal microbiota composition, increasing the relative abundance of Firmicutes by 33%, while concurrently elevating the Firmicutes to Bacteroidetes (F/B) ratio show a microbial signature strongly associated with enhanced energy harvest and superior production performance in ruminants.

Furthermore, HVP positively modulates the overall structure and functional capacity of the gastrointestinal microbiome, significantly improving alpha diversity and taxonomic richness in the gut. These microbial enhancements are paralleled by strengthened intestinal barrier integrity and mucosal immunity, contributing to a more resilient and balanced gut ecosystem. Collectively, these results underscore the potential of HVP as a natural feed additive to promote sustainable and environmentally friendly animal husbandry practices, which aligning with the global push toward antibiotic-free livestock production systems.

Importantly, this study lays the groundwork for integrative multi-omics investigations into the crosstalk between host genetics and microbiome remodeling in indigenous goat breeds. Future studies should explore how host genomic variations influence individualized responses to probiotic or herbal interventions, thereby enabling precision nutrition strategies in small ruminant farming.

## Data Availability

The data presented in the study are deposited in the NBCI SRA repository (https://www.ncbi.nlm.nih.gov/sra), accession PRJNA1372510.

## Footnotes

1
https://www.omicstudio.cn/tool
